# Phenylpropanoid- and Flavonoid-Centered Metabolic Adaptation to Continuous Cropping Stress in Ornamental Gourd

**DOI:** 10.3390/metabo16030168

**Published:** 2026-03-03

**Authors:** Hong-Yu Li, Yun-Ping Guo, Zhi-Gang Xie, Hua-Qiang Xuan, Shu-Min Wang, Xiao-Jun Wang, Wen-Wen Li, Guo-Chen Lin, Xin Hou

**Affiliations:** 1 Liaocheng Academy of Agricultural Sciences, Liaocheng 252000, China; lcsnkylihongyu@lc.shandong.cn (H.-Y.L.); lcsnkyguoyunping@lc.shandong.cn (Y.-P.G.); lcsnkyxiezhigang@lc.shandong.cn (Z.-G.X.); lcsnkyxuanhuaqiang@lc.shandong.cn (H.-Q.X.); lcsnkysys@lc.shandong.cn (X.-J.W.); lcsnkyliwenwen@lc.shandong.cn (W.-W.L.); 2 College of Food and Bio-Engineering, Beijing Vocational College of Agriculture, Beijing 102442, China; 91629@bvca.edu.cn; 3 College of Plant Protection, Shandong Agricultural University, Tai’an 271000, China

**Keywords:** continuous cropping, untargeted metabolomics, metabolic reprogramming, phenylpropanoid, flavonoid metabolism, tissue-specific differentiation

## Abstract

**Highlights:**

Untargeted metabolomics revealed a spatially organized rhizosphere–root–leaf metabolic cascade in ornamental gourd plants under continuous cropping stress, characterized by suppressed rhizosphere metabolism, reinforced root defense metabolism, and coordinated leaf-level signaling responses. Phenylpropanoid, flavonoid, amino acid, lipid, and hormone-related pathways were identified as key biochemical drivers of replanting stress adaptation, providing quantitative tissue-specific metabolite patterns that can be used as metabolic markers for continuous cropping disorders.

**What are the main findings?**

- Continuous cropping induces a clear, quantitative metabolic hierarchy across plant–soil compartments, with strong suppression of rhizosphere metabolites, pronounced activation of root-centered defense metabolism, and coordinated enrichment of signaling-related metabolites in leaves, demonstrating a rhizosphere–root–leaf metabolic cascade under replanting stress.
- Phenylpropanoid and flavonoid biosynthesis pathways emerged as central drivers of stress adaptation, showing tissue-specific accumulation patterns that link reinforced root defense with leaf-level metabolic signaling while revealing conserved core metabolites shared across the rhizosphere, roots, and leaves.

**What are the implications of the main findings?**

- The multi-compartment untargeted metabolomics framework demonstrates how environmental perturbations reorganize metabolic pathways at the system level, providing a transferable strategy for identifying spatially resolved metabolic biomarkers, pathway signatures, and metabolite networks relevant to metabolomics, systems biology, and agricultural research.
- The identification of phenylpropanoid-, flavonoid-, amino acid-, lipid-, and hormone-associated pathway reprogramming under continuous cropping stress offers metabolite-based targets for stress diagnosis and crop management, supporting the use of metabolomics to link environmental stressors with functional metabolic adaptation in plant–soil systems.

**Abstract:**

Background: Continuous cropping severely restricts ornamental gourd productivity through yield decline, microbial dysbiosis, and rhizosphere autotoxin production. This study characterized rhizosphere–root–leaf metabolic reorganization under three-year monoculture, identifying key metabolites, pathways, and a hierarchical cascade for stress adaptation. Methods: Ornamental gourd seedlings were potted in three-year monoculture soil exhibiting replanting disorders. At the seven-leaf stage, rhizosphere soil, roots, and leaves were sampled for untargeted UHPLC-MS/MS metabolomics, followed by PCA, OPLS-DA, differential analysis (VIP > 1, *p* < 0.05), and KEGG pathway enrichment analysis. Results: A total of 10,792 metabolic features were detected in positive mode and 8992 in negative mode. PCA explained 83.84% of the variance, with PC1 at 56.35% and PC2 at 27.49%, clearly separating the compartments of the study. A total of 1132 shared metabolites were suppressed, with log2 fold changes exceeding −1. Roots displayed activation, with upregulated metabolites outnumbering downregulated ones, and log2 fold changes frequently exceeding +3. Leaves exhibited mean log2 fold changes of approximately +1 for phenylpropanoid intermediates, indole, and terpenoid biosynthesis. The enriched pathways included amino acid metabolism, phenylpropanoid and flavonoid biosynthesis, lipid metabolism, and hormone signaling. Conclusions: Continuous cropping induces a hierarchical rhizosphere–root–leaf metabolic cascade, linking suppressed soil activity with reinforced root defense and coordinated leaf signaling, centered on the phenylpropanoid and flavonoid pathways as key drivers of adaptation.

## 1. Introduction

Cucurbitaceae is one of the world’s most important edible plant families, comprising approximately 95 genera and over 900 species. It ranks the fourth among plant families, following Typically, they comprise several commonly seen agronomic species, such as cucumber (*Cucumis sativus*), muskmelon (*Cucumis melo* L.), watermelon (*Citrullus lanatus*), squash/pumpkin (*Cucurbita* spp.), wax gourd (*Benincasa hispida*), luffa (*Luffa aegyptiaca*), and bitter melon (*Momordica charantia* L.), which are all recommended for use in continuous cropping systems [1,2]. Among them, *Lagenaria siceraria* (Molina) Standl., or ornamental gourd, is an economically and ecologically important crop recognized for its ornamental value and versatile agricultural applications [3]. It is widely grown by smallholder farmers for its nutritious fruit, particularly in Sub-Saharan Africa [4]. It also serves as a rootstock for grafted watermelon, improving fruit yield, quality, and resistance to Fusarium and Verticillium wilting [5,6]. In contrast to most cucurbit crops, ornamental gourds require their fruits to reach full maturity at a specific harvest stage [7]. When combined with pest and disease pressure, continuous monoculture stress can cause plants to die before maturity, resulting in severe yield losses or even complete crop failure within three consecutive years [8]. To overcome this obstacle, the sustainable cultivation of ornamental gourds in continuous cropping systems is challenging. The biophysiological mechanism underlying this phenomenon needs to be unraveled to develop fundamental strategies to cope with this issue.

Recent advances in metabolomics have highlighted the pivotal role of secondary metabolites in plant–environment interactions, particularly through root exudates and organ-specific metabolites [9,10]. These compounds play a vital role in plant defense against both biotic and abiotic stresses and influence key ornamental traits, including flavor, texture, and color [11]. Multiple studies have linked carboxylic acids, oxygenated organics, flavonoids, and polyphenols to amino acid metabolism and stress response pathways, which can improve antioxidant defenses and regulate signaling during drought and other stress conditions [12,13]. Moreover, metabolites in gourd fruits exhibit pronounced tissue-specific distributions, with substantial variations among seeds, skins, and other organs. These compounds collectively underpin their diverse physiological functions and associated health benefits, including antioxidants, antidiabetic, and anticancer activities [3,14]. In addition, the root exudates of *L. siceraria* are enriched in phenolic acids, flavonoids, and terpenoids, which can enhance plant fitness by suppressing nematodes and soil-borne pathogens, indicating an active role in shaping the rhizosphere microbiome as part of a natural defense strategy.

Despite insights into current progress, a systematic understanding of how root exudates and organ-resolved metabolism respond to continuous cropping stress remains elusive. Continuous cropping, also known as replanting syndrome, leads to a decline in yield, microbial dysbiosis, and the accumulation of autotoxins in the rhizosphere [15]. In ornamental gourd cultivation systems, continuous cropping adversely affects productivity and aesthetic quality, although the underlying biochemical mechanisms remain poorly understood [16]. Specific metabolites include cucurbitacins, organic acids, and triterpenoids, which have been reported to be associated with redox regulation, stress signaling, and tissue-specific adaptation in gourds [17,18]. However, few integrated metabolomic studies have concurrently analyzed rhizosphere, root tissue, and leaf metabolomes under continuous cropping conditions. Metabolic differentiation among rhizosphere soil, roots, and leaves represents an intrinsic feature of plant biological organization; therefore, metabolic patterns within an established continuous cropping disorder system need to be examined. A multi-compartment approach is essential for elucidating the metabolic mechanisms underlying growth inhibition caused by continuous cropping and for identifying key metabolic markers or autotoxins associated with replanting failure.

Existing research has documented stress-induced shifts in metabolite pathways that are associated with environmental perturbations. However, few studies have investigated how root-derived or tissue-specific metabolites contribute to the tolerant response to continuous cropping stress in gourd crops [19,20]. Recent studies using transcriptomic and metabolomic approaches in *L. siceraria* rootstocks have identified stress-responsive transcription factors and antioxidant pathways that may be metabolically regulated under repeated planting conditions [21]. Secondary metabolites examined in ornamental gourds include flavonoids, phenylpropanoids, and triterpenoids, which contribute to environmental adaptation by enhancing stress tolerance and physiological performance [22,23,24,25]. These compounds contribute to the synthesis of symptomatic performances, including improved antioxidant capacity, reduced water loss under drought conditions, and increased resistance to salt stress and pathogen attack. They also exhibit bioactive properties and potential health benefits [26,27]. Furthermore, root exudates have been shown to inhibit soil pathogens [28,29], indicating that gourd plants may interact with rhizosphere microbial communities through secreted metabolites as part of their natural defense system [30,31]. Despite these findings, the metabolic mechanisms underlying continuous cropping disorders remain poorly understood, despite their role as a complex syndrome that causes growth inhibition and yield decline. In particular, no comprehensive untargeted metabolomic study has concurrently profiled the rhizosphere, root, and leaf tissues of ornamental gourds under continuous cropping conditions to date. This integrated approach is essential for elucidating system-wide metabolic reprogramming, identifying stress-related metabolites or autotoxins, and revealing organ-specific responses to stress.

In this study, we aimed to characterize the spatial metabolic organization of ornamental gourds under a well-established continuous cropping disorder system, rather than directly comparing stressed versus non-stressed plants. Using multi-compartment untargeted metabolomics, we sought to describe the system-level metabolic reprogramming that occurs under real agronomic replanting conditions. The explicit aims of this study were as follows: (1) to characterize spatial metabolic differentiation across rhizosphere soil, roots, and leaves, identifying conserved core metabolites alongside strong tissue-specific specialization; (2) to identify key stress-responsive metabolites and pathways, revealing suppressed rhizosphere metabolism, reinforced root-centered defense metabolism, and coordinated leaf signaling metabolism; and (3) to integrate pathway-level insights to define a hierarchical rhizosphere–root–leaf metabolic cascade underpinning plant adaptation to continuous cropping stress. By revealing the metabolic features associated with plant–soil interactions and stress adaptation, this study provides a metabolic basis for understanding ornamental gourd responses to replanting pressure and for developing targeted strategies for crop improvement and soil health management.

## 2. Materials and Methods

### 2.1. Plant and Soil Materials

Gourd seeds used in this study originated from the Benzhang (BZ) accession, a self-bred germplasm maintained in the seed incubation chamber of the Liaocheng Academy of Agricultural Sciences, Shandong, China. Field soil samples were collected from Zhangzhuang Village, Tangyi Town, Dongchangfu District, Liaocheng City, Shandong Province, China (36.49° N, 115.78° E). The study site was located within the warm-temperate monsoon climatic zone characteristic of Shandong Province, which experiences four distinct seasons. Winters are typically cold and dry with limited precipitation, whereas summers are hot, humid, and rainy, with most of the annual rainfall occurring from June to August. The mean annual temperatures across this region of Shandong generally range from approximately 11 °C to 14 °C, and the annual precipitation varies from approximately 550 mm to 950 mm, influencing soil moisture dynamics and crop performance during the growing season. The selected field had been under continuous ornamental gourd monoculture for three consecutive years before soil sampling, and preliminary observations revealed pronounced symptoms of continuous cropping disorders, including stunted growth, reduced vigor, and uneven canopy development. These phenotypic symptoms are widely recognized as characteristic manifestations of continuous cropping (replanting) disorder in cucurbit systems; therefore, the experimental soil represents a realistic agronomic stress background rather than an artificially induced treatment. The soil management practices at the site reflected the common local agronomic routines. Annual tillage comprised conventional plowing to a depth of approximately 20–25 cm to incorporate crop residues and fertilizers. The fertilization regime predominantly involved the application of compound nitrogen (N)-phosphorus (P)-potassium (K) fertilizer (CropCare, SoneF Chemical Ltd., Qingdao, China) with an N–P_2_O_5_–K_2_O ratio of 15-15-15 at an annual rate of approximately 600 kg ha^−1^. This balanced granular fertilizer contains equal proportions of the primary nutrients N, P, and K and is widely used to support overall plant growth and development in vegetable and horticultural systems. Representative bulk soil samples were collected from the plow layer (0–25 cm) after removing surface debris and plant material had been removed. The soils were classified as typical sandy soils with moderate fertility, exhibiting a pH of 7.3, organic matter of 26.7 g kg^−1^, total N of 1.3 g kg^−1^, available P of 33.2 mg kg^−1^, and available K of 65.8 mg kg^−1^. After collecting, the soil samples were air-dried under ambient laboratory conditions to reduce moisture variability and stabilize their physicochemical properties. The dried soils were then sieved through a 2-mm mesh to remove stones, roots, and organic residues, yielding a homogenized soil material for subsequent pot experiments and untargeted metabolomics profiling.

### 2.2. Ornamental Gourd Monocultures

An ornamental gourd accession (BZ) was used in this study. The seeds of ornamental gourds were surface sterilized with 75% ethanol for 1 min, followed by 2% NaClO for 15 min, and then thoroughly washed with distilled water. The sterilized seeds were germinated on half-strength Murashige and Skoog (1/2 MS) agar-solidified medium in a growth chamber. Germinated seedlings were used in all subsequent experiments.

An experiment was conducted to detect metabolites in ornamental gourd tissues and associated samples, including rhizosphere soil (RS), root tissue (RT), and leaf tissue (LT) samples. One germinated seedling was transplanted into each plastic pot (11 cm diameter × 12 cm height) containing 800 g of soil. Soil was collected from an experimental plot that had undergone three consecutive years of ornamental gourd monoculture and exhibited clear symptoms of replanting stress. All pots were placed in a growth chamber, watered daily, and their positions were randomized weekly.

When the bottle gourd seedlings reached the seven-leaf stage, they were removed from the soil for further analysis. The roots and leaves of the harvested seedlings were subjected to untargeted metabolomic analyses. After gently shaking off loosely adhering soil as bulk soil, the roots were placed in plastic tubes for further analysis. The tubes were shaken, and the soil dislodged from the roots was collected as rhizosphere soil. Soil samples were collected for subsequent root exudate incubation, as described in the following sections.

Metabolites from the rhizosphere soil were extracted using a fresh soil extraction method. Sterilized shovels were used for soil collection, and surface impurities such as plant residues were removed prior to sampling. For rhizosphere metabolite extraction, soil adhering to 10 randomly selected plants within the same experimental block was combined to obtain sufficient material and to reduce microscale soil heterogeneity. However, each plant was grown in an individual pot and considered an independent biological unit for the statistical analysis. Three independent experimental blocks were established, and samples from each block were processed individually. In total, three independent biological replicates (blocks) were analyzed per sample type, and within each block, individual plants were used as independent biological units. No plant tissues (roots or leaves) were pooled across blocks for statistical analysis.

With three biological replicates, 100 g of fresh rhizosphere soil was collected and mixed with 500 mL deionized water. The mixture was shaken at room temperature for 3 h and centrifuged at 20 °C and 8000 rpm for 5 min. The supernatant was collected, subjected to suction filtration, and concentrated to dryness at 35 °C using a vacuum rotary evaporator. The resulting concentrate was further processed by vacuum freeze-drying (lyophilization), and the final dry powder was stored at −80 °C until subsequent analysis.

### 2.3. Metabolite Extraction

Rhizosphere soil (RS), root tissue (RT), and leaf tissue (LT) samples were prepared for metabolite extraction. A 100 mg aliquot of each solid sample was placed in a 2 mL centrifuge tube, and a 6 mm diameter grinding bead was added to it. An 800 μL volume of extraction solution (methanol:water = 4:1, *v*:*v*) containing four internal standards (0.02 mg/mL L-2-chlorophenylalanine, etc.) was added for metabolite extraction. The samples were ground using a Wonbio-96c frozen tissue grinder (Shanghai Wanbo Biotechnology Co., Ltd., Shanghai, China) for 6 min at −10 °C and 50 Hz, followed by low-temperature ultrasonic extraction for 30 min at 5 °C and 40 kHz. The samples were then incubated at −20 °C for 30 min and centrifuged at 13,000× *g* for 15 min at 4 °C. The resulting supernatants were transferred to injection vials for liquid chromatography-tandem mass spectrometry (LC-MS/MS) analysis. Quality control samples were prepared as part of the system conditioning and quality control processes. A pooled quality control (QC) sample was generated by mixing equal volumes of each sample. The quality control samples were processed and analyzed using the same procedures as the analytical samples. These quality control samples represented the entire sample set and were injected at regular intervals (every 5–15 samples) to monitor the stability and reproducibility of the analytical system.

### 2.4. UHPLC-MS/MS Analysis

LC-MS/MS analysis of the sample was conducted using a Thermo UHPLC-Q Exactive system equipped with an ACQUITY BEH C18 column (100 mm × 2.1 mm i.d., 1.7 μm; Waters, Milford, Waltham, MA, USA) (Majorbio Bio-Pharm Technology Ltd., Shanghai, China). The mobile phases consisted of 0.1% formic acid in water:acetonitrile (2:98, *v*/*v*) (solvent A) and 0.1% formic acid in acetonitrile (solvent B). The gradient conditions were as follows: 0–0.5 min, mobile phase B was maintained at 2%; 0.5–7.5 min, mobile phase B was increased from 2% to 35%; 7.5–13 min, mobile phase B was increased from 35% to 95%; 13–14.4 min, mobile phase B was maintained at 95%; 14.4–14.5 min, mobile phase B was decreased from 95% to 2%; 14.5–16 min, mobile phase B was maintained at 2%. The flow rate was 0.40 mL/min and the column temperature was 40 °C. The UPLC system was coupled to a Thermo UHPLC-Q Exactive Mass Spectrometer equipped with an electrospray ionization (ESI) source operating in positive and negative modes. The optimal conditions were as follows: source temperature, 400 °C; sheath gas flow rate, 40 arb; aux gas flow rate, 10 arb; ion-spray voltage floating (ISVF), −2800 V in negative mode and 3500 V in positive mode; normalized collision energy, 20–40–60 V rolling for MS/MS. The full MS resolution was 70,000 and the MS/MS resolution was 17,500. Data acquisition was performed using the data-dependent acquisition (DDA) mode. The detection was performed over a mass range of 70–1050 *m*/*z*.

### 2.5. Data Analysis

The LC/MS raw data were pretreated using Progenesis QI (v3.0, Waters Corporation, Milford, MA, USA) software, and a three-dimensional data matrix in CSV format was exported for further analysis. The three-dimensional matrix included sample information, metabolite names, and mass spectral response intensities. Internal standard peaks, as well as any known false-positive peaks (including noise, column bleed, and derivatized reagent peaks), were removed from the data matrix, deredundant, and peak-pooled. Simultaneously, the metabolites were identified by searching for a self-built plant-specific metabolite database (MJDBPM). The detailed annotation parameters for the key differential metabolites are presented in Table S1.

Data were analyzed using a free online platform [32]. Metabolic features detected in at least 80% of the samples were retained. After filtering, minimum metabolite values were imputed for specific samples in which the metabolite levels fell below the lower limit of quantitation, and each metabolic feature was normalized to the sum. To reduce the errors caused by sample preparation and instrument instability, the response intensity of the sample mass spectrum peaks was normalized using the sum normalization method, and a normalized data matrix was obtained. Variables with a relative standard deviation (RSD) > 30% of QC samples were removed, and log 10 processing was performed to obtain the final data matrix for subsequent analyses.

Biological replication was based on independent plants grown in separate pots within three blocks. Statistical analyses were conducted using independent plant-level replicates (n = 3 per tissue per block unless otherwise specified). The R package ‘ropls’ (Version 1.6.2) (Etienne A. Thévenot (CEA, MetaboHUB), Gif-sur-Yvette, France) was used to perform OPLS-DA. Model stability and predictive ability were evaluated using a 7-cycle interactive validation and reported as *Q*^2^ cumulative values. To further assess the risk of overfitting, a permutation test (*n* = 200) was performed for every model. Models were considered valid if *Q*^2^ > 0.5 and the *Q*^2^ regression line intercept was <0.05. Differential metabolites were selected based on Variable Importance in Projection (VIP) > 1 and *p* < 0.05 from the Student’s *t*-test.

Differential metabolites between the two groups were mapped to their biochemical pathways through metabolic enrichment and pathway analysis based on the Kyoto Encyclopedia of Genes and Genomes (KEGG) database [33]. These metabolites can be classified according to the pathways in which they are involved or the functions they perform. Enrichment analysis was used to determine whether a group of metabolites appeared in a functional node. The principle was that the annotation analysis of a single metabolite developed into an annotation analysis of a group of metabolites. The Python package “scipy.stats” (ver. 1.13.2) [34] was used to perform enrichment analysis to obtain the most relevant biological pathways for the experimental treatment.

## 3. Results

### 3.1. Primary vs. Secondary Metabolisms

**Figure 1:**
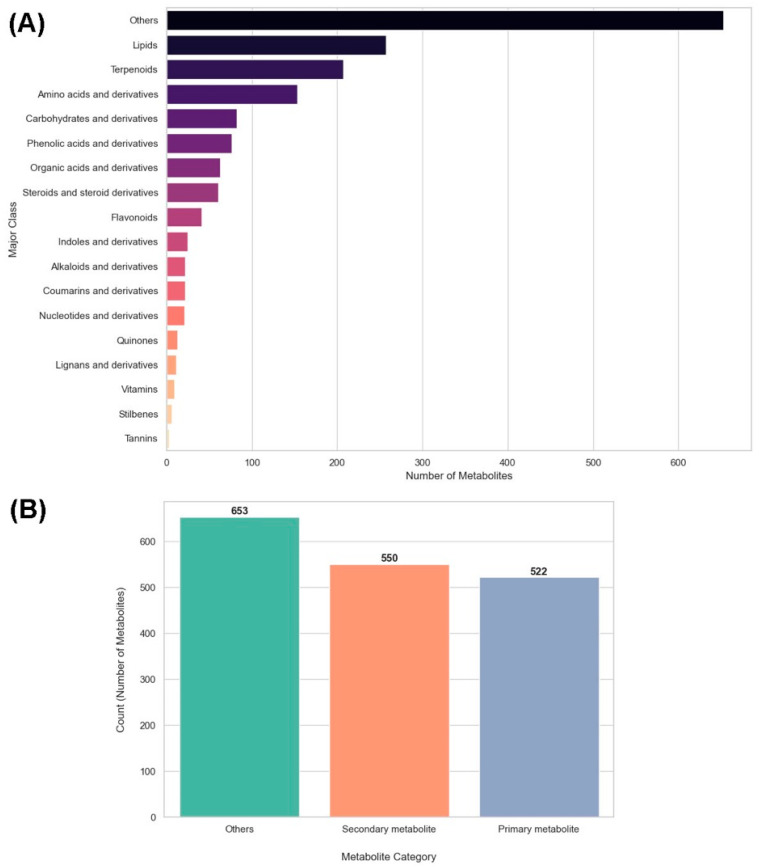
Metabolite distribution in ornamental gourds under replanting stress. (**A**) Bar chart of metabolite counts by major classes, with Others and Lipids predominating. (**B**) Bar chart comparing the counts of others (653), secondary (550), and primary (522) metabolites.

Figure 1 quantifies the metabolite composition detected under replanting stress. In Figure 1A, the “Others” class contained the largest number of metabolites (>650), exceeding the second-largest class, lipids (~260), by approximately 2.5-fold. Terpenoids (~210) and amino acids and their derivatives (~160) followed, while carbohydrates and their derivatives (~90) and phenolic acids and their derivatives (~80) showed intermediate representations. Organic acids and derivatives (~65) and steroids and steroid derivatives (~60) were less abundant, and all remaining classes individually contained fewer than 50 metabolites, with tannins representing the smallest group (<10). Figure 1B shows that metabolites categorized as “Others” totaled 653 compared to 550 secondary metabolites and 522 primary metabolites. The “Others” category exceeded secondary metabolites by 103 compounds and primary metabolites by 131 compounds, whereas secondary metabolites outnumbered primary metabolites by 28. Together, these counts indicate that non-classified metabolites constitute the largest numerical fraction relative to both primary and secondary metabolite categories.

**Figure 2:**
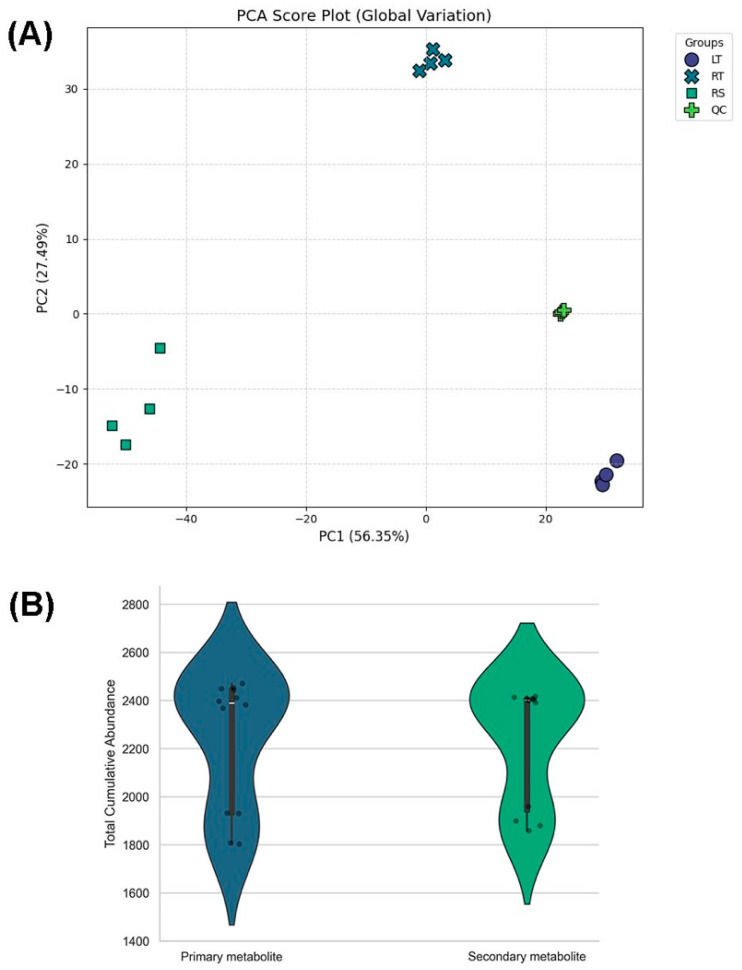
Metabolic variation in ornamental gourds under replanting stress. (**A**) PCA score plot showing global separation among the leaf (LT), root (RT), rhizosphere (RS), and quality control (QC) samples. (**B**) Violin plot of the total cumulative abundance of primary (blue) and secondary (green) metabolites.

Figure 2 shows the quantitative patterns of metabolite variation across the sample types. In Figure 2A, the first principal component (PC1) explained 56.35% of the total variance, while PC2 explained 27.49%, together accounting for 83.84% of the overall metabolic variation. The leaf (LT), root (RT), and rhizosphere (RS) samples occupied non-overlapping regions in the score plot. LT samples clustered at PC1 values of approximately +25 to +30 with PC2 near −20, RT samples clustered near PC1 ≈ 0 to +2 and PC2 ≈ +32 to +35, and RS samples clustered at PC1 values of approximately −55 to −45 with PC2 between −18 and −5. The quality control (QC) sample group was tightly clustered around PC1 ≈ +20 and PC2 ≈ 0, indicating minimal dispersion relative to the biological-sample group. In Figure 2B, the total cumulative abundance of primary metabolites ranged from approximately 1800–2450, with a median of ~2300. Secondary metabolites showed a comparable range of ~1850–2400, with a similar median (~2300). The interquartile ranges of the two metabolite categories largely overlapped, and both distributions showed comparable spreads in abundance values.

**Figure 3:**
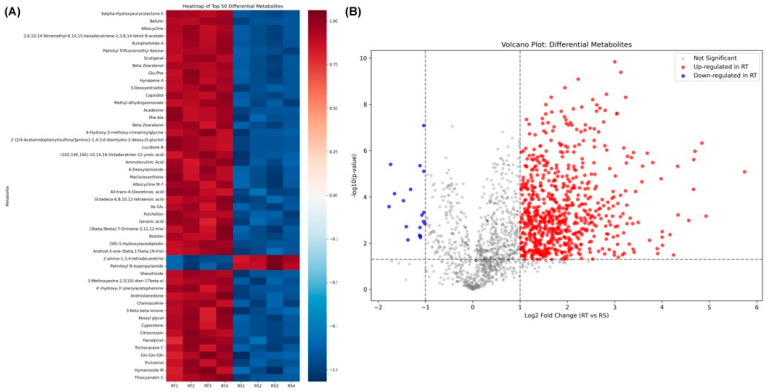
Metabolic profiling of ornamental gourd under replanting stress. (**A**) Heatmap of the top 50 differential metabolites across the root (RT) and rhizosphere (RS) samples, showing clustering based on expression levels. (**B**) Volcano plot depicting log2 fold changes (RT vs. RS) and significance, with red (upregulated) and blue (downregulated) dots.

Figure 3 summarizes the quantitative differences in metabolite abundance between the root (RT) and rhizosphere (RS) samples. As shown in Figure 3A, hierarchical clustering of the top 50 differential metabolites showed consistent within-group similarity across the four replicates per tissue. Standardized abundance values were predominantly positive (approximately +0.6 to +1.1) in the RT and negative (approximately −0.6 to −1.1) in the RS for most metabolites, indicating opposite relative scaling between the two compartments across all 50 features. Only a small subset displayed an inverse pattern, with higher scaled values in the RS than in the RT. In Figure 3B, the volcano plot applied log_2_ fold change and −log_10_(*p*-value) thresholds of ±1 and ~1.3, respectively. A substantially larger number of metabolites fell on the positive side of the *x*-axis, with log_2_ fold changes ranging from ~1 to >5, and −log_10_(*p*-values) frequently exceeding 2 and reaching above 9. Fewer metabolites showed negative log_2_ fold changes below −1, and these downregulated features were comparatively limited in number and significance. The numerical distribution indicated that the significantly increased metabolites in RT outnumbered the significantly decreased metabolites under the applied statistical criteria.

**Figure 4:**
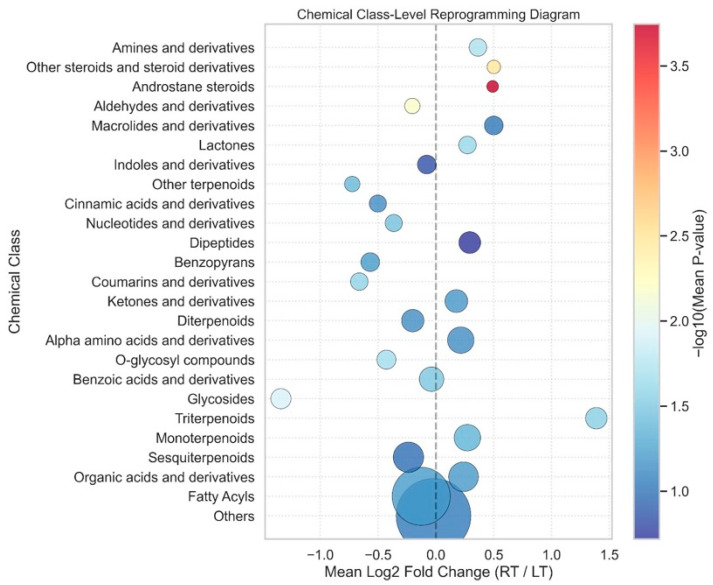
Chemical class-level reprogramming diagram illustrating spatial metabolic shifts in ornamental gourds under the stress of continuous replanting. The bubble plot displays the mean log2 fold change (root/leaf) on the *x*-axis and −log10(mean *p*-value) on the *y*-axis (color gradient from blue to red indicating increasing significance), highlighting the differential abundance of chemical classes, such as amines, steroids, terpenoids, amino acids, and others, between the root and leaf tissues. Data represent n = 3 biological replicates for the rhizosphere, with root and leaf tissues pooled from 10 independent plants post-measurements to maintain biological independence and obtain sufficient material for analysis.

Figure 4 quantifies the class-level differences between the root and leaf tissues using the mean log_2_ fold change and aggregated statistical support. Several classes showed positive shifts (RT/LT > 0), including monoterpenoids (~0.35), diterpenoids (~0.25), alpha-amino acids and derivatives (~0.30), ketones and derivatives (~0.20), and macrolides and derivatives (~0.40). Among these, androstane steroids displayed one of the highest positive mean fold changes (~0.45) and the strongest significance signal (−log_10_P ≈ 3.6). In contrast, multiple classes were negatively shifted, notably fatty acyls (≈−0.05), organic acids and derivatives (≈−0.15), sesquiterpenoids (≈−0.10), and glycosides (≈−1.10). The glycoside class exhibited a large negative fold change with moderate significance (−log_10_P ≈ 1.7). The bubble size indicates that “Others” and fatty acyls accounted for the largest number of metabolites, but clustered near zero or negative fold change, with relatively low to moderate significance. Overall, the quantitative dispersion across classes spanned approximately −1.2 to +1.4 in the mean log_2_ fold change, with statistical strength varying independently of class size.

**Figure 5:**
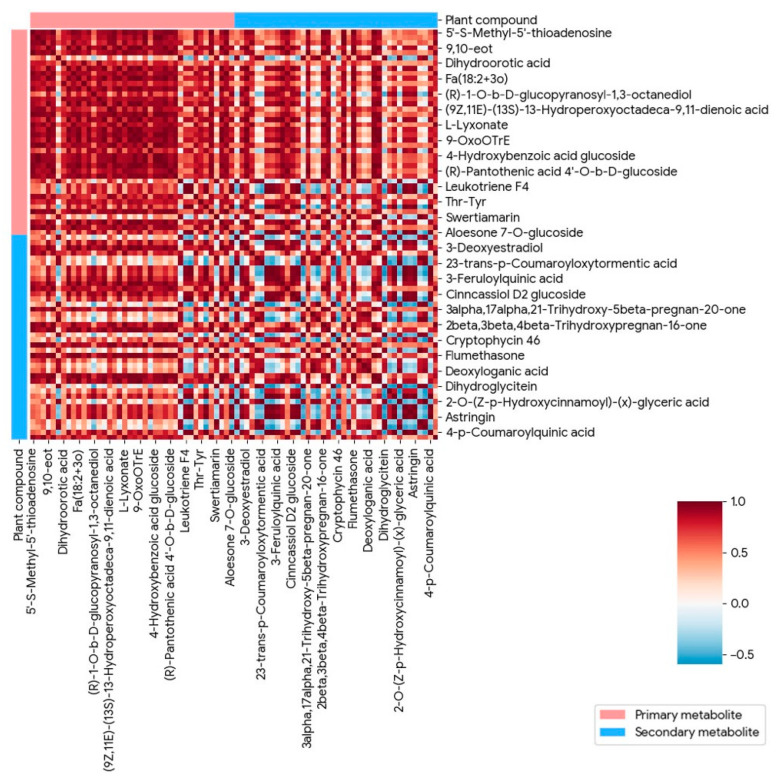
Correlation heatmap illustrating the relationships between primary (pink) and secondary (blue) metabolites in ornamental gourds under continuous cropping stress, based on untargeted metabolomic profiling of rhizosphere–root–leaf tissues.

Across the primary–secondary metabolite correlation matrix in Figure 5, the pairwise Pearson correlation coefficients ranged from approximately −0.5 to 1.0, with the majority of coefficients clustering above 0.3. More than two-thirds of the metabolite pairs exhibited positive correlations exceeding 0.4, and a substantial subset showed strong positive associations in the range of 0.7–1.0, indicating tight quantitative coupling. Negative correlations were comparatively limited in number and magnitude, with most inverse relationships ranging from −0.2 to −0.5. Primary–primary and secondary–secondary metabolite pairs displayed similar correlation distributions, but cross-category (primary–secondary) correlations accounted for the highest frequency of coefficients above 0.6. Several metabolite pairs reached near-maximal correlation values close to 1.0, whereas only a small fraction approached the lower bound. Overall, the numerical structure of the matrix was dominated by moderate to strong positive correlations, with weak or negative associations representing a minor proportion of the total pairwise comparisons.

### 3.2. Phenylpropanoid Biosynthesis Pathway

**Figure 6:**
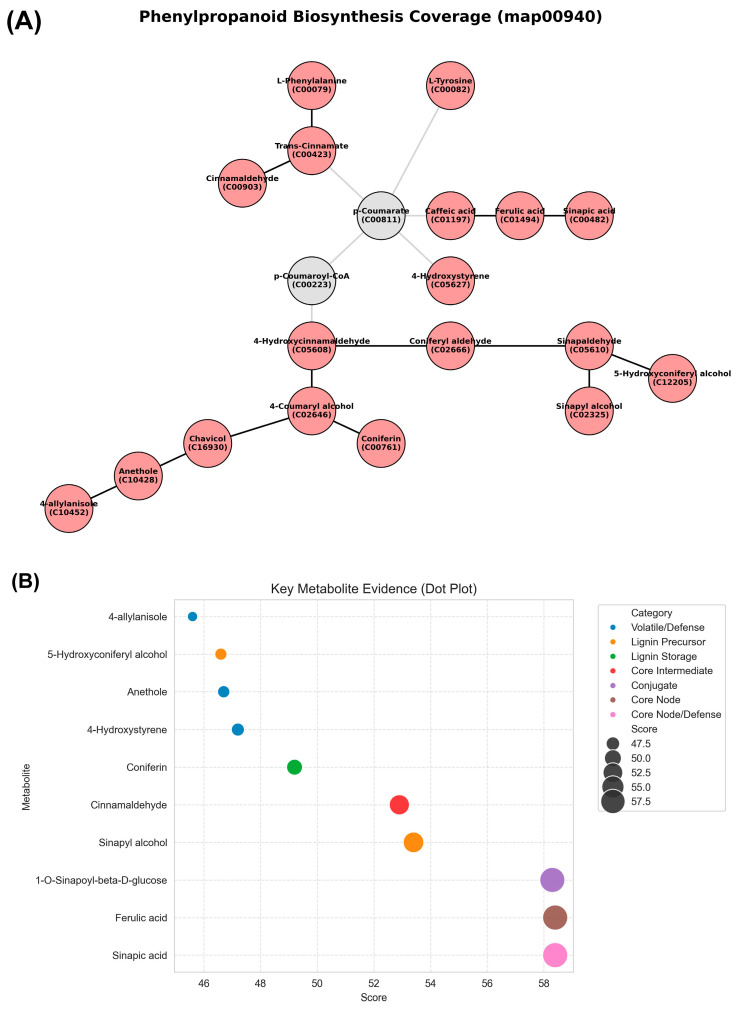
Phenylpropanoid biosynthesis pathway (KEGG map00940) in ornamental gourds under replanting stress, showing metabolic network coverage and key metabolite evidence scores. (**A**) Network map illustrating phenylpropanoid biosynthesis coverage, with nodes representing metabolites. (**B**) Dot plot of key metabolite evidence scores (46–58) categorized by function.

Figure 6 quantifies the metabolite representation and evidence strength of the phenylpropanoid pathway. In Figure 6A, 14 metabolites were mapped onto the KEGG phenylpropanoid biosynthesis network, spanning upstream amino acid precursors, intermediate aldehydes, acids, alcohols, and downstream conjugates of phenylpropanoid biosynthesis. Most of the detected nodes corresponded to phenylpropanoid intermediates derived from phenylalanine or tyrosine, whereas a smaller number represented activated CoA esters and glycosylated products. Therefore, pathway coverage includes multiple consecutive reaction steps rather than isolated nodes. As shown in Figure 6B, the key metabolite evidence scores ranged from approximately 46–58. Volatile/defense-related compounds (4-allylanisole, anethole, and 4-hydroxystyrene) showed lower scores clustered between ~46 and 48. Coniferin exhibited an intermediate score near ~50. Core intermediates and lignin-related metabolites exhibited higher scores, including cinnamaldehyde (~52), sinapyl alcohol (~54), and ferulic acid (~57) scores. The highest scores were observed for sinapic acid and 1-O-sinapoyl-β-D-glucose, both approaching ~58. The numerical distribution indicates a gradient of evidence strength across the different compound categories.

### 3.3. Flavonoid Biosynthesis Pathway

**Figure 7:**
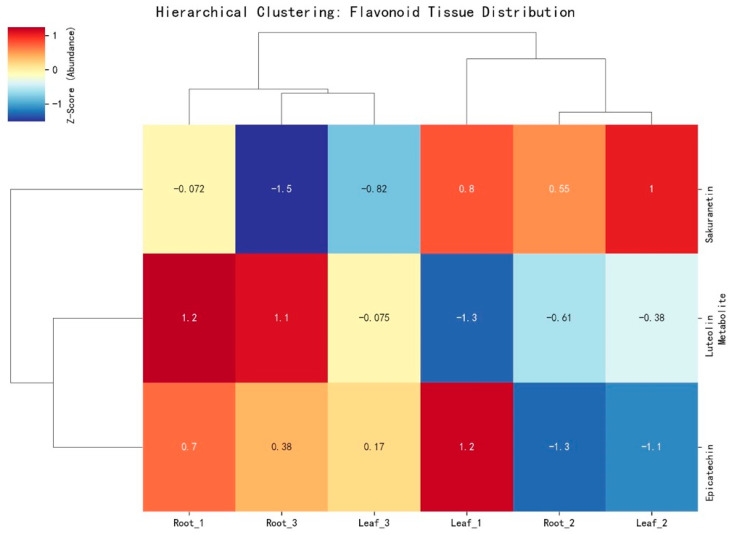
Hierarchical clustering heatmap illustrating Z-score-normalized abundance of flavonoids (sakuranetin, luteolin, epicatechin) across the root and leaf tissues in ornamental gourd under replanting stress, revealing spatial metabolic differentiation.

Figure 7 shows the Z-score-normalized abundance values of the three flavonoids in the root and leaf samples. Sakuranetin showed negative Z-scores in roots (−0.07 to −1.50) and positive values in leaves (0.55 to 1.00), with the highest value at Leaf_2 (1.00). Luteolin displayed the opposite pattern, with higher Z-scores in roots (1.10–1.20) and lower values in leaves (−1.30 to −0.38). Epicatechin exhibited positive Z-scores in Root_1 (0.70) and Leaf_1 (1.20) but negative values in Root_2 (−1.30) and Leaf_2 (−1.10), indicating a greater dispersion across samples. Hierarchical clustering grouped samples with similar Z-score ranges, separating those with Z-scores above 0.5 from those below −1.0. The numerical spread across metabolites ranged from −1.50 to +1.20, indicating a total normalized variation span of approximately 2.7 units among tissues and replicates.

The roots contained three identified metabolites, representing 60% of the total (3/5), whereas the rhizosphere and leaves contained one metabolite each (20% each). The phytochemical subclass composition was evenly distributed, with O-methylated flavonoids, flavones, and flavonols accounting for 33.3% of the total subclass.

**Figure 8:**
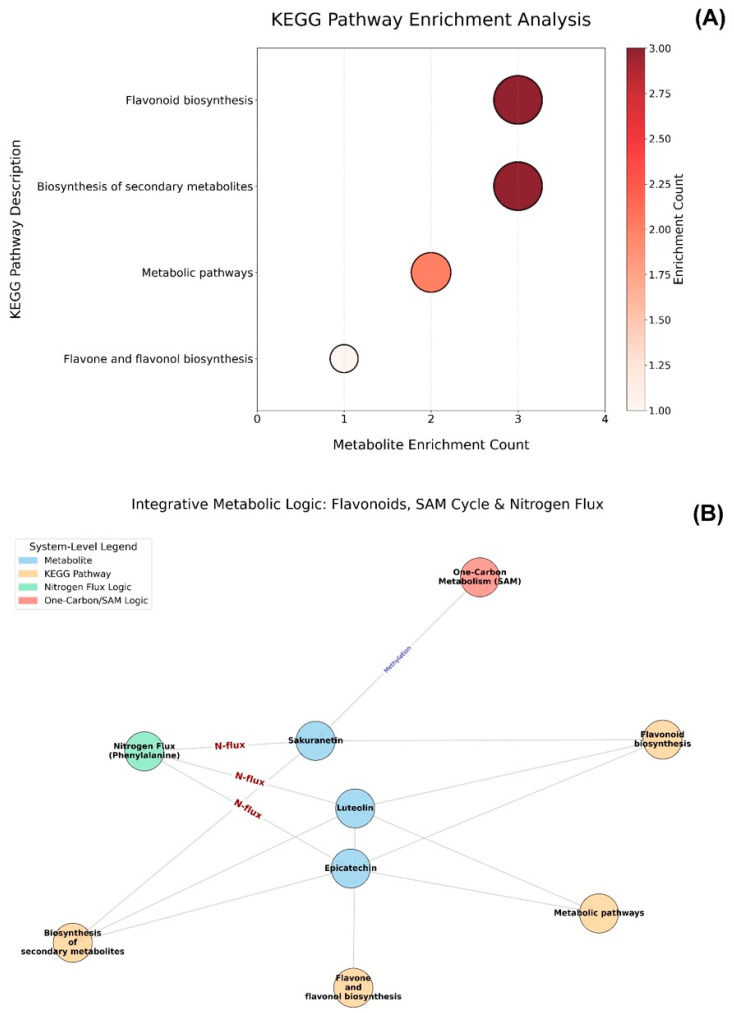
KEGG pathway enrichment and integrative metabolic logic in ornamental gourd under replanting stress. (**A**) Bubble plot of enriched pathways, with the *x*-axis showing metabolite enrichment count, the *y*-axis listing pathways (e.g., flavonoid biosynthesis), bubble size proportional to count, and color gradient indicating the enrichment level. (**B**) Network diagram depicting connections among flavonoids, the SAM cycle, nitrogen flux, and related pathways, with nodes colored according to category.

Figure 8 summarizes the pathway-level enrichment counts and the network connectivity. In Figure 8A, flavonoid biosynthesis and the biosynthesis of secondary metabolites each showed an enrichment count of three, representing the highest counts among the pathways listed. Metabolic pathways showed an intermediate enrichment count of 2, whereas flavone and flavonol biosynthesis showed an enrichment count of one. Thus, pathways with enrichment counts ≥2 accounted for 75% (three of four) of the enriched pathways displayed. As shown in Figure 8B, the integrative network included three metabolites (sakuranetin, luteolin, and epicatechin), four KEGG pathways, one nitrogen flux node, and one one-carbon/SAM node, totaling nine nodes. Sakuranetin was associated with three system-level nodes (nitrogen flux, one-carbon metabolism, and flavonoid biosynthesis), whereas luteolin and epicatechin were each associated with three KEGG pathways. The network comprised ten edges, with metabolite nodes accounting for the majority of pathway-level connections based on the link counts shown.

### 3.4. Inter-Tissue Metabolite Changes

**Figure 9:**
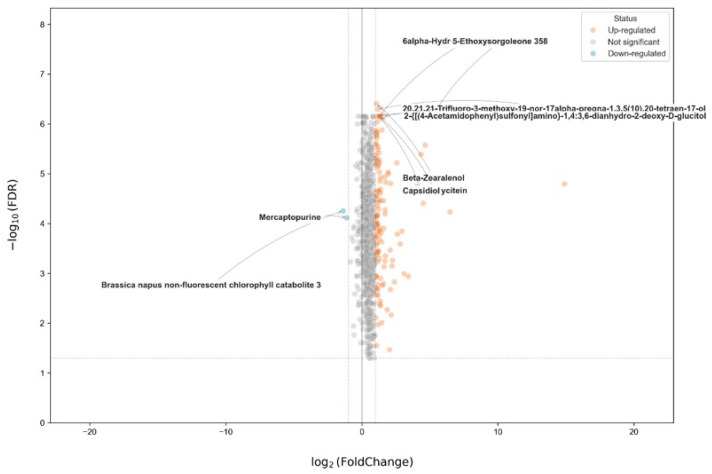
Volcano plot of differential metabolites in ornamental gourds under replanting stress (RB vs. EB). The *x*-axis shows log_2_(fold change), and the *y*-axis shows –log_10_(FDR). Points are colored by regulation status: upregulated (orange), downregulated (blue), and not significant (gray). The selected key metabolites are labeled in the figure.

Figure 9 shows the quantitative differences in metabolite abundance between the RB and EB groups. The *x*-axis spanned log_2_ fold changes from approximately −22 to +21, whereas the *y*-axis (−log_10_ FDR) extended to approximately 8. Upregulated metabolites were predominantly distributed on the positive side of the log_2_ fold-change axis, with many exceeding +1 and several exceeding +10. Among the annotated points, multiple metabolites showed −log_10_(FDR) values between ~5 and ~7, corresponding to FDR values < 10^−5^. Downregulated metabolites were fewer and mainly clustered between log_2_ fold changes of −1 and −5, with limited points extending beyond −10. Their −log_10_(FDR) values generally ranged from approximately 3 to 4.5. Most detected features fell near log_2_ fold change values between −1 and +1 and below the horizontal significance threshold (~1.3 on the −log_10_ scale), indicating non-significant differences between the groups. Overall, the number of significantly upregulated metabolites exceeded that of significantly downregulated metabolites under the applied thresholds.

## 4. Discussion

### 4.1. Metabolic Composition and Global Metabolic Variation

The global metabolic landscape revealed by untargeted profiling suggests stress-associated metabolic reprogramming under continuous cropping conditions, reorganizing the rhizosphere–root–leaf continuum into a spatially differentiated system. Clear multivariate separation among compartments, together with the presence of a conserved core metabolite pool, is consistent with earlier tissue-resolved metabolomic studies showing that plant stress responses preserve basal metabolism while reallocating specialized compounds to specific organs [10,11]. The pronounced contraction of amines, nucleobase derivatives, and lipid-associated metabolites in the rhizosphere mirrors the metabolic suppression reported in other replanting and monoculture systems, where reduced exudate diversity weakens microbial functional activity and intensifies soil sickness [15,30].

In contrast, the roots exhibited a marked shift toward amino acids, organic acids, and phenylpropanoid-derived metabolites, indicating a defense-oriented metabolic state in the roots. Similar accumulation of flavonoids and lignans has been widely documented in cucurbit roots and other crops exposed to chronic soil stress, where these compounds contribute to antioxidant buffering and structural reinforcement rather than growth promotion [17,21,23]. Such root-centered metabolic intensification supports the view that primary and secondary metabolism are tightly coupled under continuous cropping, thereby maintaining redox balance and internal energy supply during prolonged stress [11].

Leaf metabolism displayed a contrasting pattern characterized by the enrichment of phenylpropanoid intermediates, indoles, and terpenoids, which aligns with reports that leaves function as integrative signaling hubs during root-derived stress rather than terminal defense sites [3,35]. Comparable increases in leaf terpenoids and upstream phenylpropanoid compounds have been associated with the systemic coordination of carbon allocation and stress perception in cucurbit and woody plants [36,37]. Collectively, these compartment-specific shifts support a hierarchical metabolic cascade in which rhizosphere suppression, root defense reinforcement, and leaf-level coordination operate in concert rather than as uniform whole-plant responses under continuous cropping stress. While inherent compartmental differentiation contributes to the baseline metabolic structure, the magnitude of log_2_ fold changes, the quantitative imbalance between upregulated and downregulated metabolites, and the coordinated enrichment of defense-related pathways collectively suggest stress-amplified remodeling rather than simple tissue specialization.

### 4.2. Phenylpropanoid and Flavonoid Pathways as Drivers of Stress Adaptation

Primary and secondary metabolisms exhibit clearly differentiated yet coordinated responses to continuous cropping stress across the rhizosphere–root–leaf system. Primary metabolic pathways related to amino acid turnover and central carbon flux show pronounced spatial adjustment, particularly in the roots, where the enhanced accumulation of basic amino acids supports basal growth maintenance under replanting pressure. Similar reinforcement of amino acid metabolism has been reported in stressed cucurbit rootstocks and other crops exposed to monoculture fatigue, where nitrogen reallocation preserves essential physiological functions despite environmental constraints [15,19]. In contrast, rhizosphere soils display a suppression of primary metabolites, such as amines and nucleobase derivatives, which is consistent with the reduced microbial and biochemical activity observed in long-term monocropping systems [16].

Secondary metabolism exhibits stronger tissue specificity and predominantly contributes to stress mitigation rather than growth support. Roots preferentially enrich flavonoids and related phenylpropanoid intermediates, which aligns with earlier observations that root-localized secondary metabolites act as chemical barriers against soil-borne pathogens and autotoxin accumulation [25,38]. However, leaves favor the accumulation of downstream phenylpropanoids, indoles, and terpenoids, indicating a shift toward systemic signaling and physiological coordination rather than localized defense deployment. Comparable leaf-centered increases in signaling-associated secondary metabolites have been documented in gourd and non-gourd species exposed to abiotic and replanting stresses, where photosynthetic tissues integrate metabolic cues from belowground organs [24,39].

The divergence between primary metabolic stabilization and secondary metabolic amplification reflects a functional trade-off rather than a metabolic imbalance. Previous metabolomic studies have suggested that plants under chronic stress prioritize secondary metabolite biosynthesis once minimal primary metabolic demands are secured, enhancing resilience without fully restoring growth potential [10,11,22]. In this context, the present findings support a hierarchical metabolic model in which primary metabolism maintains structural viability, whereas secondary metabolism orchestrates adaptive responses across interconnected plant compartments.

### 4.3. Phenylpropanoid- and Flavonoid-Biosynthesis Pathway

The phenylpropanoid and flavonoid biosynthesis pathways were markedly activated across the rhizosphere–root–leaf continuum, indicating their central role in metabolic adaptation to continuous cropping. In the root and leaf tissues, the enrichment of phenylpropanoid intermediates and diverse flavonoids is consistent with the stress-associated redirection of carbon flux toward secondary metabolism, a pattern that has been widely reported in plants exposed to adverse environments [40,41]. Metabolite identification was confirmed by cross-referencing with public databases such as KEGG and HMDB, achieving MSI Level 2 based on accurate mass (errors typically <5 ppm) and high MS/MS fragmentation scores (>85.0 on average for key compounds). For example, Table S1 in the Supplementary Materials details the confirmation metrics for representative phenylpropanoid- and flavonoid-related metabolites, such as ferulic acid (KEGG: C01494, mass error: −0.91 ppm, fragmentation score: 96.9) and sinapic acid (KEGG: C00482, mass error: −2.02 ppm, fragmentation score: 97.0). Similar reinforcement of these pathways has been observed under abiotic stresses, such as drought, salinity, and temperature fluctuations, where phenylpropanoid-derived compounds contribute to antioxidative capacity and cell wall fortification [42,43]. Compared to studies on woody and herbaceous species, the elevated accumulation of flavonoids in root tissues aligns with evidence that roots act as primary sites for the deployment of defensive metabolites, particularly under soil-derived stress [44,45]. At the leaf level, increased phenylpropanoid and flavonoid metabolites resemble responses reported in foliage subjected to light- or pathogen-induced oxidative pressure, where these compounds function as ROS scavengers and signaling mediators rather than terminal defense products [46,47].

Notably, the coordinated enhancement of both phenylpropanoid and flavonoid biosynthesis mirrors the findings of integrated metabolomic–transcriptomic studies, which demonstrated that the simultaneous activation of PAL-, 4CL-, and CHS-related steps underpins effective stress tolerance [48,49]. Compared with nutrient- or pollutant-induced responses, where pathway activation often exhibits strong tissue specificity, the concurrent enrichment observed here across multiple compartments suggests a systemic metabolic adjustment driven by continuous cropping pressure [50,51]. Collectively, these results support the view that phenylpropanoid and flavonoid metabolism constitutes a conserved yet plastic defense module, enabling ornamental gourds to integrate belowground stress perception with aboveground physiological coordination under replanting stress conditions [52,53].

### 4.4. Tissue-Specific Metabolite Variation

The marked separation of metabolite profiles among rhizosphere soil, roots, and leaves indicates a spatially organized metabolic strategy rather than a uniform stress response to salinity. Root tissues preferentially accumulate flavonoids, lignans, and amino acid-derived metabolites, a pattern widely reported in plants in which roots function as primary defensive and sensing organs under adverse conditions [54,55]. Similar root-biased enrichment of phenylpropanoid end products has been linked to strengthened local defense and the modulation of soil interactions in medicinal and crop species [56]. In contrast, leaves displayed elevated levels of phenylpropanoid intermediates and diverse terpenoids, which aligns with reports that aerial tissues maintain metabolic flexibility to coordinate systemic signaling and redox balance under stress [57,58]. This partition supports the view that leaves emphasize metabolic preparedness, whereas roots prioritize terminal defensive synthesis.

Comparative transcriptome–metabolome studies have further demonstrated that tissue-specific metabolite accumulation often reflects the differential regulation of biosynthetic genes rather than substrate limitations [59,60]. Regulatory control by tissue-biased transcription factors, particularly R2R3-MYB members, drives flavonoid and phenylpropanoid allocation among organs [37]. Consistent with these observations, coordinated amino acid and nitrogen-related metabolites in roots suggest a reprogrammed nitrogen flux that supports secondary metabolism during persistent stress [61]. Collectively, comparisons with previous studies indicate that tissue-specific metabolite variation represents an adaptive hierarchy in which roots deploy localized chemical defenses and leaves integrate systemic metabolic signals, thereby optimizing whole-plant resilience to chronic environmental pressure.

### 4.5. Study Limitations and Future Directions

This study has three limitations that can be addressed in future work.

First, the present study was conducted within a field-derived continuous cropping disorder system and did not include first-year planting or non-continuously cropped soil as a parallel control group. Therefore, while the observed metabolic patterns are strongly associated with replanting stress under real agronomic conditions, future comparative experiments incorporating non-stressed controls will be necessary to fully distinguish baseline tissue differentiation from stress-amplified metabolic remodeling and enable strict causal inference.

Second, although the spatial metabolite patterns and correlation analyses were consistent with a directed metabolic cascade, functional experiments (e.g., transcriptomics, enzyme activity measurements, or isotope-labeled tracing) are needed to confirm causality and distinguish between interconnected signaling and independent tissue responses. Such experiments could include the targeted gene expression analysis of key pathway enzymes (e.g., in phenylpropanoid and flavonoid biosynthesis) and metabolic flux studies to verify the directionality of the proposed cascade.

Finally, this study relied exclusively on untargeted metabolomics without integrating transcriptomic or proteomic validation. Although metabolite-level changes reveal pathway reprogramming, the absence of multi-omics confirmation limits the mechanistic interpretation of gene regulation and enzyme activity. Consequently, the causal relationships between metabolic shifts and stress adaptation remain inferential rather than experimentally validated.

## 5. Conclusions

The methodology applied an untargeted, multi-compartment metabolomic framework that integrated rhizosphere soil, root tissue, and leaf tissue profiling using UHPLC-MS/MS, multivariate statistics, and pathway-level network analysis, which enabled the robust characterization of spatial metabolic organization under continuous cropping stress. The major results showed that continuous cropping stress drove pronounced tissue-specific metabolic differentiation, characterized by suppressed rhizosphere metabolite abundance, reinforced root-centered accumulation of flavonoids and amino acid-related metabolites, and coordinated leaf-level enrichment of signaling-associated intermediates, indicating a structured rhizosphere–root–leaf metabolic cascade. Taken together, these findings suggest that continuous cropping stress reorganizes metabolism into a hierarchical rhizosphere–root–leaf cascade, in which suppressed soil biochemical activity may be coupled with root-centered defense reinforcement and leaf-level coordination. This structured metabolic response suggests that spatial compartmentalization is the dominant strategy underlying stress adaptation in ornamental gourds. Future studies should incorporate non-stressed controls, temporal sampling across growth stages, and functional validation experiments to clarify the causal relationships between specific metabolites, microbial interactions, and plant performance under continuous cropping conditions.

## Data Availability

The dataset will be available upon request from the authors. The raw data supporting the conclusions of this article will be made available by the authors upon request.

## Supplementary Materials

The following supporting information can be downloaded at: https://www.mdpi.com/article/10.3390/metabo16030168/s1, Table S1: Detailed annotation parameters for key differential metabolites. Figure S1: Permutation test validation plots for OPLS-DA models. The statistical validity of the OPLS-DA models was evaluated using 200-cycle permutation tests. In each panel, the horizontal axis represents the correlation between the permuted class labels and the original labels, and the vertical axis represents the R2Y (green circles) and Q2 (blue squares) values. The regression lines for R2Y and Q2 are represented by dashed lines. (a) Rhizosphere soil versus root tissue; (b) root tissue versus leaf tissue; (c) rhizosphere soil versus leaf tissue.

## Abbreviations

The following abbreviations are used in this manuscript:

Abbreviation	Full Term
4CL	4-Coumarate-CoA ligase
BZ	Benzhang accession
CHS	Chalcone synthase
CoA	Coenzyme A
DDA	Data-Dependent Acquisition
ESI	Electrospray Ionization
FDR	False Discovery Rate
ISVF	Ion-Spray Voltage Floating
K	Potassium
KEGG	Kyoto Encyclopedia of Genes and Genomes
LC-MS/MS	Liquid Chromatography-Tandem Mass Spectrometry
LT	Leaf Tissue
MJDBPM	Self-Built Plant-Specific Metabolite Database
MS	Murashige and Skoog
MYB	MYB Family Transcription Factor (R2R3-MYB in plants)
N	Nitrogen
OPLS-DA	Orthogonal Partial Least Squares Discriminant Analysis
P	Phosphorus
PAL	Phenylalanine ammonia-lyase
PC1	First Principal Component
PC2	Second Principal Component
PCA	Principal Component Analysis
QC	Quality Control
RS	Rhizosphere Soil
RSD	Relative Standard Deviation
RT	Root Tissue
SAM	S-Adenosylmethionine
UHPLC	Ultra-High-Performance Liquid Chromatography
VIP	Variable Importance in the Projection

## References

[1] Li Z., Zhu J., Shi T., Gao C., Qiu X., Bao M., Li Y., Bi Z., Yao P., Sun C. (2026). Microbial community restructuring under crop rotation: A sustainable strategy to counteract potato monoculture-induced soil degradation in arid ecosystems. Agric. Ecosyst. Environ..

[2] Schwacke R., Bolger M.E., Usadel B. (2025). PubPlant—A continuously updated online resource for sequenced and published plant genomes. Front. Plant Sci..

[3] Bhutani M., Gaur S.S., Shams R., Dash K.K. (2025). Sustainable utilization of bottle gourd by-products: A nutritional and functional perspective. Food Chem. Adv..

[4] Mkhize P., Shimelis H., Mashilo J. (2025). Hybrid performance of bottle gourd [Lagenaria siceraria] under drought stress and non-stress conditions. Ecol. Genet. Genom..

[5] Suárez-Hernández A.M., Grimaldo-Juárez O., Ceceña-Durán C., Vázquez-Angulo J.C., Carrazco-Peña L.D., Avendaño-Reyes L., Ail-Catzim C.E., Basilio-Cortes U.A., Angulo-Castro A. (2022). Influence of Seed and Fruit Characteristics of Lagenaria siceraria on Production and Quality of Grafted Watermelon. Horticulturae.

[6] Aras V., Sarı N., Solmaz İ. (2022). Effects of Cucurbita, Lagenaria and Citrullus rootstocks on pollen and fruit characters, seed yield and quality of F1 hybrid watermelon. Int. J. Agric. Environ. Food Sci..

[7] Dhaliwal M.S., Pessarakli M. (2017). CHAPTER 5 Cucurbits. Handbook of Cucurbits: Growth, Cultural Practices, and Physiology.

[8] Tang L., Hamid Y., Chen Z., Lin Q., Shohag M.J.I., He Z., Yang X. (2021). A phytoremediation coupled with agro-production mode suppresses Fusarium wilt disease and alleviates cadmium phytotoxicity of cucumber (*Cucumis sativus* L.) in continuous cropping greenhouse soil. Chemosphere.

[9] Martins S., Brito C., Baltazar M., Dinis L.-T., Pereira S. (2026). Exploring the Role of Root Exudates in Shaping Plant–Soil–Microbe Interactions to Support Agroecosystem Resilience. Horticulturae.

[10] Jacoby R.P., Chen L., Schwier M., Koprivova A., Kopriva S. (2020). Recent advances in the role of plant metabolites in shaping the root microbiome. F1000Research.

[11] Arbona V., Manzi M., Ollas C.D., Gómez-Cadenas A. (2013). Metabolomics as a Tool to Investigate Abiotic Stress Tolerance in Plants. Int. J. Mol. Sci..

[12] Nayak J.K.K., Panda B., Mahapatra D., Mohanty D., Mondal S., Mohanty A., Sangeeta S., Nayak G., Senapaty J., Panda B., Aires A. (2025). Flavonoids: A Natural Shield of Plants under Drought Stress. Plant Secondary Metabolites—Occurrence, Structure and Role.

[13] Patil J.R., Mhatre K.J., Yadav K., Yadav L.S., Srivastava S., Nikalje G.C. (2024). Flavonoids in plant-environment interactions and stress responses. Discov. Plants.

[14] Li J., Liu H., Xu Y., Yang J., Yu Y., Wen J., Xie D., Zhong Y., Wu J., Fu M. (2025). Metabolomic Analysis of Different Parts of Black Wax Gourd (*Cucurbita pepo*). Foods.

[15] Cao S., Li Y., Dong J., Qin B., Yang G., Yin Y., Zhao W. (2025). Decline in rhizosphere VOC diversity drives microbiome restructuring inducing *Fritillaria pallidiflora* replant disease. Ind. Crops Prod..

[16] Xu J., Zhang N.Y., Wang K., Xian Q.Q., Dong J.P., Chen X.H. (2022). Exploring new strategies in diseases resistance of horticultural crops. Front. Sustain. Food Syst..

[17] Maja D., Mavengahama S., Mashilo J. (2022). Cucurbitacin biosynthesis in cucurbit crops, their pharmaceutical value and agricultural application for management of biotic and abiotic stress: A review. S. Afr. J. Bot..

[18] Flores-Iga G., Lopez-Ortiz C., Natarajan P., Nimmakayala P., Reddy U.K., Balagurusamy N., Almeida A. (2025). Cucurbitacin Profile and Metalloid Stress Response in *Cucurbita pepo* L. Upon Arsenic Exposure. Plant Direct.

[19] Zeeshan Ul Haq M., Yu J., Yao G., Yang H., Iqbal H.A., Tahir H., Cui H., Liu Y., Wu Y. (2023). A Systematic Review on the Continuous Cropping Obstacles and Control Strategies in Medicinal Plants. Int. J. Mol. Sci..

[20] Zhang F., Rosental L., Ji B., Brotman Y., Dai M. (2024). Metabolite-mediated adaptation of crops to drought and the acquisition of tolerance. Plant J..

[21] Liu J., Zhang M., Xu J., Yao X., Lou L., Hou Q., Zhu L., Yang X., Liu G., Xu J. (2024). A Transcriptomic Analysis of Bottle Gourd-Type Rootstock Roots Identifies Novel Transcription Factors Responsive to Low Root Zone Temperature Stress. Int. J. Mol. Sci..

[22] Ninkuu V., Aluko O.O., Yan J.P., Zeng H.M., Liu G.D., Zhao J., Li H.H., Chen S., Dakora F.D. (2025). Phenylpropanoids metabolism: Recent insight into stress tolerance and plant development cues. Front. Plant Sci..

[23] Chen D.L., Mubeen B., Hasnain A., Rizwan M., Adrees M., Naqvi S.A.H., Iqbal S., Kamran M., El-Sabrout A.M., Elansary H.O. (2022). Role of Promising Secondary Metabolites to Confer Resistance Against Environmental Stresses in Crop Plants: Current Scenario and Future Perspectives. Front. Plant Sci..

[24] Tang H., Wang Q., Xie H., Li W. (2024). The function of secondary metabolites in resisting stresses in horticultural plants. Fruit Res..

[25] Liu F., Yang J., Mu H., Li X., Zhang X., Wen Y., Zhang X. (2023). Effects of Brassinolide on Growth, Photosynthetic Rate and Antioxidant Enzyme Activity of Ornamental Gourd under Salt Stress. Russ. J. Plant Physiol..

[26] Ramaroson M.-L., Koutouan C., Helesbeux J.-J., Le Clerc V., Hamama L., Geoffriau E., Briard M. (2022). Role of Phenylpropanoids and Flavonoids in Plant Resistance to Pests and Diseases. Molecules.

[27] Ullah A., Munir S., Badshah S.L., Khan N., Ghani L., Poulson B.G., Emwas A.-H., Jaremko M. (2020). Important Flavonoids and Their Role as a Therapeutic Agent. Molecules.

[28] Hauer R.J., Wei H., Koeser A.K., Dawson J.O. (2021). Gas Exchange, Water Use Efficiency, and Biomass Partitioning among Geographic Sources of Acer saccharum Subsp. saccharum and Subsp. nigrum Seedlings in Response to Water Stress. Plants.

[29] Wei H.X., Xu C.Y., Ren J., Ma L.Y., Duan J., Jiang L.N. (2013). Newly transplanted *Larix olgensis* Henry stock with greater root biomass has higher early nitrogen flux rate. Soil Sci. Plant Nutr..

[30] Afzal M.R., Naz M., Yu Y., Yan L., Wang P., Mohotti J., Hao G., Zhou J.-J., Chen Z., Zhang L. (2025). Root exudates: The rhizospheric frontier for advancing sustainable plant protection. Resour. Environ. Sustain..

[31] Yang Z., Niu J.Z., Wu T., Li J.Q., Zhang L., Chen X.W., Berndtsson R. (2025). Impact of root exudates on soil reconstruction and bacterial community resumption in open-pit coal mines. Front. Microbiol..

[32] Majorbio Majorbio: A Provider of One-Stop, Hassle-Free Omics Solutions. https://cloud.majorbio.com.

[33] KEGG KEGG: Kyoto Encyclopedia of Genes and Genomes. https://www.genome.jp/kegg/.

[34] SciPy SciPy Documentation. https://docs.scipy.org/doc/scipy/.

[35] Dueñas M.E., Klein A.T., Alexander L.E., Yandeau-Nelson M.D., Nikolau B.J., Lee Y.J. (2017). High spatial resolution mass spectrometry imaging reveals the genetically programmed, developmental modification of the distribution of thylakoid membrane lipids among individual cells of maize leaf. Plant J..

[36] Liu J., Gao Y., He Z.Q., Zhang H., Chen L.J. (2023). The efficacy of sodium bicarbonated Ringer’s solution versus lactated Ringer’s solution in elderly patients undergoing gastrointestinal surgery: A prospective randomized controlled trial. Am. J. Transl. Res..

[37] Huang H.P., Yang X.N., Yang Z.R. (2025). Integration of transcriptome and metabolome analysis reveals the genes and pathways regulating flavonoids biosynthesis in Cinnamomum camphora. BMC Genom. Data.

[38] Chen J., Shi Y.L., Zhong Y.C., Sun Z.M., Niu J., Wang Y., Chen T.X., Chen J., Luan M. (2022). Transcriptome Analysis and HPLC Profiling of Flavonoid Biosynthesis in *Citrus aurantium* L. during Its Key Developmental Stages. Biology.

[39] Zhang P., Li J., Li T., Li X.X., Lu Y., Wu J.W. (2024). Transcriptome analysis of potassium-mediated cadmium accumulation in sweet sorghum. Plant Physiol. Biochem..

[40] Amjadi Z., Hamzehzarghani H., Rodriguez V.M., Huang Y.J., Farahbakhsh F. (2024). Studying temperature’s impact on Brassica napus resistance to identify key regulatory mechanisms using comparative metabolomics. Sci. Rep..

[41] Bocaj V., Pongrac P., Fischer S., Likar M. (2024). Species-Specific and Pollution-Induced Changes in Gene Expression and Metabolome of Closely Related Noccaea Species Under Natural Conditions. Plants.

[42] Chen M.M., Yang Y.Y., Han X., Nie G.P., Li X., Wang Z., Cai Y.M., Yang L., Zhang Y. (2024). Metabolomics integrated with transcriptomics provides insights into the phenylpropanoids biosynthesis pathway in *Lilium davidii* var. *unicolor* and *L. lancifolium* Thunb. Int. J. Biol. Macromol..

[43] Chen X.F., Jiang C., Long M.Q., Hu X.X., Xu S.H., Huo H.T., Shi R.X., Xu Q., Xie S., Li Z. (2025). Overexpression of the Glycyrrhiza uralensis Phenylalanine Ammonia-Lyase Gene GuPAL1 Promotes Flavonoid Accumulation in Arabidopsis thaliana. Int. J. Mol. Sci..

[44] Chang E., Guo W., Dong Y., Jia Z.R., Zhao X.L., Jiang Z.P., Zhang L., Zhang J., Liu J. (2023). Metabolic profiling reveals key metabolites regulating adventitious root formation in ancient Platycladus orientalis cuttings. Front. Plant Sci..

[45] Chen Q., Luo L.X., Zhou T., Gan J.X., Liu N.F., Lu R., Xu Q., Hu L., Chen G. (2025). Comparative Transcriptome Analysis of Leaves and Roots Revealed Organ-Specific and Cross-Stress Defense Strategies of Pearl Millet Under Different Abiotic Stresses. Agronomy.

[46] Bin Y., Zhang Q., Su Y., Wang C.Q., Jiang Q.Q., Song Z., Zhou C.Y. (2023). Transcriptome analysis of Citrus limon infected with Citrus yellowvein clearing virus. BMC Genom..

[47] Chen H.Q., Deng L.Z., Yang B.W., Chang X.X., Chen Z., Qiu J.S., Peng C., Lu Y. (2026). Integrated multi-omics reveals the mechanisms of sunburn-induced peel browning in wampee fruit. Plant Physiol. Biochem..

[48] Arshad M., Ma Y.W., Gao W.C., Zhang S.X., Shoaib M., Liu X.R., Fan Y.K., Li G., Chuai H., Jiang Y. (2025). Polypropylene microplastic exposure modulates multiple metabolic pathways in tobacco leaves, impacting lignin biosynthesis. Ecotoxicol. Environ. Saf..

[49] Cai D., Dong Y.J., Wang L., Zhao S.C. (2025). Integrated metabolomics and transcriptomics analysis provides insights into biosynthesis and accumulation of flavonoids and glucosinolates in different radish varieties. Curr. Res. Food Sci..

[50] Chen S.P., Chen Z.Q., Zhuang Q.Q., Chen H.W. (2025). Multi-omics joint analysis reveals the mechanism of flower color and fragrance variation in *Lilium cernuum*. Front. Plant Sci..

[51] Chen L.L., Xu Z.L., He Y.Q., Zhang X.Y., Li L.Y., Zhu R.F., Zhang Z.L., Lin H., Hong G. (2025). Multiomics Analysis Reveals Key Targeted Metabolic Pathways Underlying the Hormesis and Detrimental Effects of Enrofloxacin on Rice Plants. J. Agric. Food Chem..

[52] An L., Yuan Y.L., Chen H., Li M., Ma J.W., Zhou J., Zheng L.F., Ma H., Chen Z., Hao C. (2024). Comprehensive widely targeted metabolomics to decipher the molecular mechanisms of Dioscorea opposita thunb. cv. Tiegun quality formation during harvest. Food Chem.-X.

[53] Cao H., Ding L.Z., Yu C., Zhao K.L., Zhao W.M., Fang X.Z., Ma J.W., Liu D., Ye Z. (2024). Sensitivity of Chinese Hickory to Soil Acidification and Important Plant Metabolites in Response to Soil Acidification. Pol. J. Environ. Stud..

[54] Fakhrah S., Bano N., Sarvendra K., Lone R.A., Nayak S.P., Kumari A., Rout P.K., Mohanty C.S. (2025). Elucidating the Secondary Metabolite Biosynthesis Networks in Underutilized Tree Bean (*Parkia timoriana*) Through Integrated Metabolomic and Transcriptomic Approaches. Appl. Biochem. Biotechnol..

[55] Huang J.F., Qin Y.L., Xie Z.L., Wang P., Zhao Z.C., Huang X.L., Chen Q.F., Huang Z., Chen Y., Gao A. (2023). Combined transcriptome and metabolome analysis reveal that the white and yellow mango pulp colors are associated with carotenoid and flavonoid accumulation, and phytohormone signaling. Genomics.

[56] Ma Q., Wang S.D., Tan H.T., Sun Z.K., Li C.W., Zhang G.Y. (2024). Tissue-specific transcriptome analyses unveils candidate genes for flavonoid biosynthesis, regulation and transport in the medicinal plant Ilex asprella. Sci. Rep..

[57] Aguilar-Méndez E.D., Monribot-Villanueva J.L., Guerrero-Analco J.A., De-la-Peña C. (2024). Chlorophyll deficiency in Agave angustifolia Haw.: Unveiling the impact on secondary metabolite production. Planta.

[58] Martinez-Alonso A., Yepes-Molina L., Guarnizo A.L., Carvajal M. (2023). Modification of Gene Expression of Tomato Plants through Foliar Flavonoid Application in Relation to Enhanced Growth. Genes.

[59] Chen F.Q., Ha X., Ma T., Ma H.L. (2024). Comparative analysis of the physiological and transcriptomic profiles reveals alfalfa drought resistance mechanisms. BMC Plant Biol..

[60] Gao Y.Y., Lai J.L., Feng C.L., Li L.Y., Zu Q.H., Li J., Du D.X. (2025). Transcriptional Analysis of Tissues in Tartary Buckwheat Seedlings Under IAA Stimulation. Genes.

[61] Kim G., Sung J. (2023). Transcriptional Expression of Nitrogen Metabolism Genes and Primary Metabolic Variations in Rice Affected by Different Water Status. Plants.

