# Supplementary material for: Phenylpropanoid- and Flavonoid-Centered Metabolic Adaptation to Continuous Cropping Stress in Ornamental Gourd
Source: Metabolites. 2026 Mar 3;16(3):168. doi: 10.3390/metabo16030168 (PMC13027611; doi:10.3390/metabo16030168)
Supplement: Supplementary file 1 [file metabolites-16-00168-s001.zip › metabolites-4171494-supplementary.pdf]

Table S1. Detailed annotation parameters for key differential metabolites.

Metabolite Name	KEGG ID	HMDB ID	Mass Error (ppm)	Fragmentation Score	MSI Level
Ferulic acid	C01494	HMDB0000954	-0.91	96.9	Level 2
Sinapic acid	C00482	HMDB0032616	-2.02	97	Level 2
Caffeic acid	C01481	HMDB0001101	-1.15	95.8	Level 2
Trans-Cinnamate	C00423	-	-1.57	85.6	Level 2
Sinapyl alcohol	C02325	HMDB0013070	-1.4	72.2	Level 2
Cinnamaldehyde	C00903	HMDB0003441	-0.91	68.4	Level 2
Epicatechin	C09727	HMDB0001871	-9.02*	43	Level 2
Sakuranetin	C09833	HMDB0030090	0.75	62.2 (Theo.)	Level 2
Luteolin	C01514	HMDB0000580	-0.66	46.1 (Theo.)	Level 2
Isoferulic acid	C10470	HMDB0000955	-3.95	93.5	Level 2

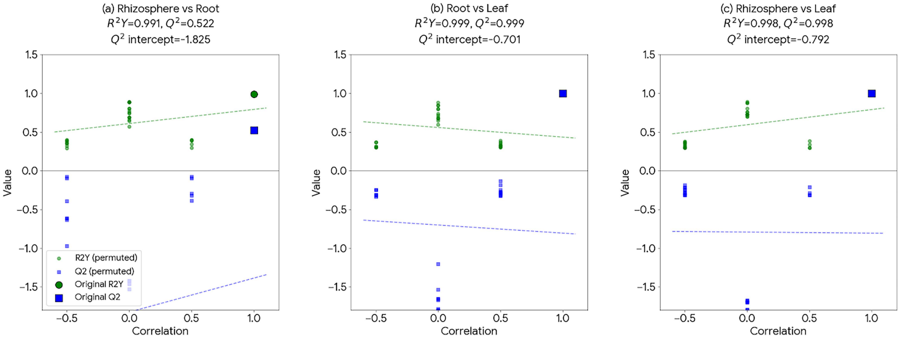
(a) Rhizosphere vs Root
R^2Y=0.991, Q^2=0.522
Q^2 intercept=-1.825
(b) Root vs Leaf
R^2Y=0.999, Q^2=0.999
Q^2 intercept=-0.701
(c) Rhizosphere vs Leaf
R^2Y=0.998, Q^2=0.998
Q^2 intercept=-0.792
Value
Correlation
R2Y (permuted)
Q2 (permuted)
Original R2Y
Original Q2
1.5
1.0
0.5
0.0
−0.5
−1.0
−1.5
−0.5
0.0
0.5
1.0
